# Differential X-Ray Attenuation in MA-XRF Analysis for a Non-invasive Determination of Gilding Thickness

**DOI:** 10.3389/fchem.2020.00175

**Published:** 2020-03-13

**Authors:** Sergio Augusto Barcellos Lins, Stefano Ridolfi, Giovanni Ettore Gigante, Roberto Cesareo, Monica Albini, Cristina Riccucci, Gabriella di Carlo, Andrea Fabbri, Paolo Branchini, Luca Tortora

**Affiliations:** ^1^Department of Basic and Applied Sciences for Engineering, Sapienza University of Rome, Rome, Italy; ^2^Surface Analysis Laboratory, Istituto Nazionale di Fisica Nucleare Sezione di Roma Tre, Rome, Italy; ^3^Ars Mensurae srl, Rome, Italy; ^4^Istituto di Matematica e Fisica, Università degli Studi di Sassari, Sassari, Italy; ^5^Institute for the Study of Nanostructured Materials, National Research Council, Rome, Italy; ^6^Department of Sciences, Roma Tre University, Rome, Italy

**Keywords:** gilding, MA-XRF, differential attenuation, Cu-based artifacts, thickness estimation

## Abstract

When investigating gilded artifacts or works of art, the determination of the gilding thickness plays a significant role in establishing restoration protocols or conservation strategies. Unfortunately, this is done by cross-sectioning the object, a destructive approach not always feasible. A non-destructive alternative, based on the differential attenuation of fluorescence radiation from the sample, has been developed in the past years, but due to the intrinsic random nature of X-rays, the study of single or few spots of an objects surface may yield biased information. Furthermore, considering the effects of both porosity and sample inhomogeneities is a practice commonly overlooked, which may introduce systematic errors. In order to overcome these matters, here we propose the extrapolation of the differential-attenuation method from single-spot X-ray fluorescence (XRF) measurements to macro-XRF (MA-XRF) scanning. In this work, an innovative algorithm was developed for evaluating the large amount of data coming from MA-XRF datasets and evaluate the thickness of a given overlapping layer over an area. This approach was adopted to study a gilded copper-based buckle from the sixteenth to seventeenth century found in Rome. The gilded object under investigation was also studied by other analytical techniques including scanning electron microscopy coupled with energy dispersive spectroscopy (SEM-EDS). Previous results obtained from SEM-EDS were used to confront the data obtained with the proposed methodology and validate it. MA-XRF elemental distribution maps were fundamental in identifying and choosing sampling areas to calculate the thickness of the gilding layer, avoiding lead islands present in the sample that could negatively influence the results. Albeit the large relative standard deviation, the mean thickness values fell within those found in literature and those obtained from previous studies with SEM-EDS. Surface fissure has been found to deeply affect the results obtained, an aspect that is often disregarded.

## Introduction

The practice of gilding dates back from antiquity and is still performed until today by means of different techniques, as electrochemical deposition instead of the toxic mercury fire-gilding (Anheuser, 1997). With the course of time, a wide array of gilding methods has been developed and those most widespread in antiquity were the application of a gold foil (or leaf) to a prepared substrate, mercury gilding, or depletion (Lechtman et al., 1982; Ingo et al., 2013; Pessanha et al., 2019b). The method chosen also depends on the type of substrate used, which varies from leather, plaster and wood to metals (Cesareo, 2003; Eveno et al., 2014; Tortora et al., 2014; Biocca et al., 2016; Shabunya-Klyachkovskaya et al., 2017; Iorio et al., 2019). In the case of fire-gilding, a mixture made of mercury and gold (amalgam) is applied to a clean metallic substrate and fired in temperatures around 300°C to volatilize the mercury. The surface is then finished with the use of burnishers and the resulted thickness can range from 1 μm to about 10 μm or more (Anheuser, 1997; Ingo et al., 2013).

Due to the variety of gilding methods used in the past and their intrinsic variations (amalgam composition and thickness), the investigation of archaeological gilded objects has raised interest in the community, aiming to investigate the methodology and materials used and the conservation state of these objects (Abdelhamid et al., 2010; Ingo et al., 2016, 2018; Graziani et al., 2020). The most straightforward way of studying a gilded objects' characteristics and gilding method is by analyzing a cross-section of the object. This approach yields direct information of the thickness of the gold layer and the binding mechanism between gold layer and substrate. Scanning electron microscopy coupled with energy dispersive spectroscopy (SEM-EDS) goes even further, giving a direct information on the chemical composition of the substrate and the gilding layer (Ingo et al., 2016). Nevertheless, the study of cross-sections requires sampling and is a destructive method, being rarely feasible when it comes to valuable artifacts (Nørgaard, 2017).

The focus on non-destructive techniques has always been a priority in cultural heritage science, and X-rays fluorescence (XRF) is commonly used as a standard approach (Guerra, 2000; Bottaini et al., 2017). This technique can provide information on the chemical composition of a sample surface and even the thickness of an existing overlapping layer—giving the conditions are optimal (van Espen, and Lemberge, 2000; Giurlani et al., 2019). Nonetheless, archaeological artifacts are known to be extremely inhomogeneous and the analysis of only few spots of the sample's surface may lead to misleading conclusions (Cesareo et al., 2010; Brunetti et al., 2016). Extrapolating the one-dimensionality of XRF technique to another dimension, i.e., scanning [or capturing in full field (Romano et al., 2014)] a surface instead of analyzing few spots, is a practice that is now becoming widespread in cultural heritage science and is generally known as macro-XRF scanning (MA-XRF) (Dik et al., 2008). This approach increases significantly the understanding of the sample, by generating bi-dimensional maps of elemental distribution and providing a large XRF dataset to work with.

Measuring the thickness of a given surface layer placed to protect an artifact or, as in the case of gilding, to turn it more attractive, can be performed by a method that uses the differential attenuation of fluorescence radiation coming from the internal matrix (substrate) (Cesareo et al., 2015). This method is based on the concatenated effect of two distinct processes: (a) the production of fluorescence photons in the internal matrix and (b) the differential attenuation in the surface layer. The latter can become quite complex when dealing with amalgam gilded objects, since the chemical composition of the surface layer is not constant throughout the sample and micro-fissures can be present in amounts sufficient to introduce significant errors. Moreover, the metallic substrate of archaeological artifacts can present considerable inhomogeneities in their composition, caused by either smelting, molding, or working. Inhomogeneities in the sample hinders a precise estimation of the matrix un-attenuated signal and therefore must be avoided. Furthermore, the presence of protective layers, such as mica or paraloids, must be cautiously accounted. The existence of an “extra” layer on top of the gilding layer further attenuates the photons coming from the substrate and therefore introduce systematic errors. If the protective layer is known, as in the case of paraloids, the further attenuation introduced can be calculated and Equation (5) can be adjusted accordingly (Nardes et al., 2019). Past interventions or cleaning routines applied to the objects surface can be assessed by the complete absence of soil impurities in the elemental distribution maps generated with the MA-XRF data. Therefore, a proper interpretation of these maps and the assessment of the presence or not of additional layers are crucial for selecting suitable regions where to apply the differential attenuation method.

In this context, the current research proposes the use of MA-XRF scan datasets to investigate the gilding technique employed in a Sixteenth to Seventeenth century AD buckle found during the dredging of Tiber river (Rome, Italy), by applying the differential attenuation method and calculating the gilding thickness in a totally non-invasive manner. The use of MA-XRF elemental distribution maps is crucial to select suitable regions from the sample surface where to calculate the thickness. Moreover, an algorithm capable of analyzing thousands of *spectra* and simulating the chemical composition of the overlapping layer, considering the presence of fissures or pores, was developed to account for the variations present in this layer. This allows a more precise calculation of the superficial layer attenuation coefficients. The method dismisses any need for cross-sectioning, sampling or even sample preparation, being completely non-destructive. Nonetheless, the buckle investigated has been previously studied by SEM-EDS (Ingo et al., 2018), where a sectioning of a small part in the rightmost portion of the object was made. Results from SEM-EDS analysis have been used to validate the proposed methodology.

## Materials and Methods

A gilded Cu-based buckle dating from the Sixteenth to Seventeenth century found during the dredging of the Tiber river in Rome was analyzed by scanning electron microscopy (SEM) coupled with energy dispersive spectroscopy (EDS) and by macro X-ray fluorescence (MA-XRF) scanning. The object was cleaned with distilled water followed by ethanol rinsing. No conservation treatment was performed. SEM images were used to confront the data obtained from the differential attenuation calculations and assess the reliability and quality of the data.

To obtain the most representative data, the thickness calculations were performed in specific regions of the sample in a way to avoid great geometrical variations and matrix composition inhomogeneities. The intensity ratio of Cu-K_α_ and Cu-K_β_ characteristic lines for an infinitely thick matrix were experimentally measured in the central portion of the sample (where no gold cover is visible), being the mean value obtained with 6 different spots.

### The Differential Attenuation Method

**Figure 1:**
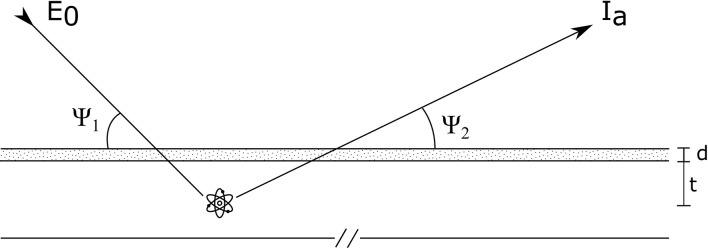
Representative scheme of impinging and outgoing radiations.

Considering one of the two monochromatic radiations used in the differential attenuation method (for example the K_α_ line of one of the elements present in the internal matrix) and a generic tube-sample-detector geometry (Figure 1), the following equation is valid for the photons detected in the spectral window a (I_a_):

(1) $$\begin{array}{l} {\text{I}}_{\text{a}}={\text{I}}_{0\;}\text{AK}\cdot \varepsilon ({\text{E}}_{\text{a}})\cdot {\text{e}}^{-(\mu_{\text{L}}({\text{E}}_{0})\text{ cos }\Psi_{1}+\mu_{\text{L}}({\text{E}}_{\text{a}})\text{ cos }\Psi_{2})\text{d}}\\ \;\int_{0}^{t}{\text{e}}^{-[\mu_{\text{s}}({\text{E}}_{0})]\text{ cos }\Psi_{1}+\;\mu_{\text{s}}({\text{E}}_{\text{a}})]\text{ cos }\Psi_{2\;}]\text{x}}\cdot \text{dx} \end{array}$$

By integrating the equation above one obtains:

(2) $$\begin{array}{l} {\text{I}}_{\text{a}}={\text{I}}_{0\;}\text{AK}\cdot \varepsilon ({\text{E}}_{\text{a}})\cdot \frac{1-{\text{e}}^{-[\mu_{\text{s}}({\text{E}}_{0})\text{ cos }\Psi_{1}+\mu_{s}({\text{E}}_{\text{a}})\text{ cos }\Psi_{2}]\text{t}}}{\begin{array}{c} \mu_{\text{s}}({\text{E}}_{0})\text{ cos }\Psi_{1}+\mu_{\text{s}}({\text{E}}_{\text{a}})\text{ cos }\Psi_{2}\\ \cdot {\text{e}}^{-(\mu_{\text{L}}({\text{E}}_{0})\text{ cos }\Psi_{1}+\mu_{\text{L}}({\text{E}}_{\text{a}})\text{ cos }\Psi_{2})]\text{d}} \end{array}} \end{array}$$

where I_0_ is the exciting photons beam intensity at energy E_0_, A is the overall term taking into account all the factors that determine the production of photons detected in the spectral window a, K is the geometrical factor, ε(E_a_) is the detector efficiency, μ_L_(E_0_) and μ_L_(E_a_) are the superficial layer attenuation coefficients at impinging and outgoing photon energies, respectively, d represents its thickness, μ_s_(E_0_) and μ_s_(E_a_) are the attenuation coefficients of the internal matrix, Ψ_1_ and Ψ_2_ are the incident beam and output angles with the sample surface, respectively and *t* is the internal matrix thickness.

The ratio between two monochromatic radiations can then be written, assuming that the detector efficiency and geometrical factors are the same for both lines, as:

(3) $$\left(\frac{{\text{I}}_{\text{a}}}{{\text{I}}_{\text{b}}}\right)={\left(\frac{{\text{I}}_{\text{a}}}{{\text{I}}_{\text{b}}}\right)}_{\text{thin}}\cdot \frac{\mu_{\text{s}}({\text{E}}_{0})\text{ cos }\Psi_{1}+\mu_{\text{s}}({\text{E}}_{\text{b}})\text{ cos }\Psi_{2}}{\begin{array}{c} \mu_{\text{s}}({\text{E}}_{0})\text{ cos }\Psi_{1}+\mu_{\text{s}}({\text{E}}_{\text{a}})\text{ cos }\Psi_{2}\\ \cdot \chi \cdot {\text{e}}^{[-\mu_{\text{L}}({\text{E}}_{\text{a}})\;+\;[\mu_{\text{L}}({\text{E}}_{\text{b}})]\text{ cos}\Psi_{2}\text{d}} \end{array}}$$

where $\chi =\;\frac{1-e^{-\left[\mu_{s}\left(E_{0}\right)\cos\left(\Psi_{1}\right)\;+\mu_{s}\left(E_{a}\right)\cos\left(\Psi_{2}\right)\;\right]t}\;}{1-e^{-\left[\mu_{s}\left(E_{0}\right)\cos\left(\Psi_{1}\right)\;+\mu_{s}\left(E_{b}\right)\cos\left(\Psi_{2}\right)\;\right]t}}$

The first term (referred to as thin) is the ratio when the self-attenuation in the internal matrix is completely negligible, the second and third terms consider the contribution of this effect and the fourth term considers the differential attenuation undergone in the surface layer. Only the fourth term depends on the surface layer, while the remaining terms depend only on the internal matrix. When the exponential contributions in the third term (χ) are negligible (having *t* large enough that the term can be approximated to 1), the sample can be assumed infinitely thick.

When the two lines are relative to a single element, as in the case of the lines K_α_ and K_β_ of copper, their ratio is fixed and well-known (Cesareo et al., 2009).

Neglecting the differential attenuation in the superficial layer and considering an infinitely thick approximation, the matrix ratio can be given by the following equation:

(4) $${\left(\frac{{\text{I}}_{\text{a}}}{{\text{I}}_{\text{b}}}\right)}_{\text{thick}}={\left(\frac{{\text{I}}_{\text{a}}}{{\text{I}}_{\text{b}}}\right)}_{\text{thin}}\cdot \frac{\mu_{\text{s}}\left({\text{E}}_{0}\right)\cos\Psi_{1}{+\mu }_{\text{s}}\left({\text{E}}_{\text{b}}\right)\cos\Psi_{2}}{\mu_{\text{s}}\left({\text{E}}_{0}\right)\cos\Psi_{1}{+\mu }_{\text{s}}\left({\text{E}}_{\text{a}}\right)\cos\Psi_{2}}$$

The values for infinitely thin samples can be calculated from the fundamental parameters (Cesareo et al., 2010), and therefore—if the sample composition is known—one can calculate the intensity ratio for a thick sample. This value can also be experimentally determined by directly measuring the sample exposed substrate or by creating a calibration model via Monte Carlo simulations (Pessanha et al., 2019a) when the former is not feasible.

Therefore, assuming the attenuation coefficient of the overlapping layer is known (its composition is known or experimentally determined using the acquired XRF data) and that the matrix can be considered infinitely thick, the superficial layer thickness is finally given by Equation (5) below (Cesareo et al., 2016; Nardes et al., 2019):

(5) $$d=\frac{sin\Psi_{2}\;}{-\mu_{L}\left(E_{K_{\alpha }}\right)+\mu_{L}\left(E_{K_{\beta }}\right)}\ln\left[\frac{\left(\frac{I_{K_{\alpha }}}{I_{K_{\beta }}}\right)}{{\left(\frac{I_{K_{\alpha }}}{I_{K_{\beta }}}\right)}_{thick}}\right]$$

### MA-XRF Scanning

The system used to record and process the XRF data was a *custom-made* portable scanner, made by the *Istituto Nazionale di Fisica Nucleare (INFN)—Roma Tre Division*, and a private company *Ars Mensurae*. This system has been employed in previous studies (Iorio et al., 2019) and comprises a movable stage—where the sample is positioned—and an exchangeable head composed of a collimated Moxtek® Ta-target X-ray tube and an AMPTEK® 123SDD detector. The spot size is of roughly 1 mm^2^ and the total scanned area was of 34 × 15 mm^2^, enough to cover the entirety of the sample. The tube operated at 37 KV and 17 μA. The dwell-time per pixel was of 7 s, resulting in roughly 60 min of acquisition time. The system was calibrated using an SRM 1115 NIST Standard reference material with the following composition expressed as mass fraction, in %: Copper 87.96, Zinc 11.73, Lead 0.013, Iron 0.13, Tin 0.10, Nickel 0.074, and Phosphorus 0.05.

### SEM-EDS

Backscattered images and Field-emission (FE) SEM images were acquired with a SEM Stereoscan 360 system from Cambridge, UK, equipped with a LaB_6_ filament and a LEO Gemini 1530 microscope from Zeiss, Germany. The former coupled to an INCA 250 and the latter to an INCA 450 energy-dispersive X-ray spectrometers (EDS) both by Oxford Instruments Analytical, UK. The images were collected with acceleration voltages up to 20 kV.

Prior to the analysis, the sample cross-section was coated with either C or Cr. This procedure is required to avoid charging effects. Carbon was deposited with an Emitech sputter coater model K550, a K250 carbon coating attachment and a carbon cord. The carbon film deposited was ~3 nm thick. As for the chromium film, the deposition procedure was performed with a Bal-Tech SCD 500 at a 5 × 10^−3^ mbar pressure to ensure a constant thickness of about 0.5 nm.

## Results and Discussion

**Figure 2:**
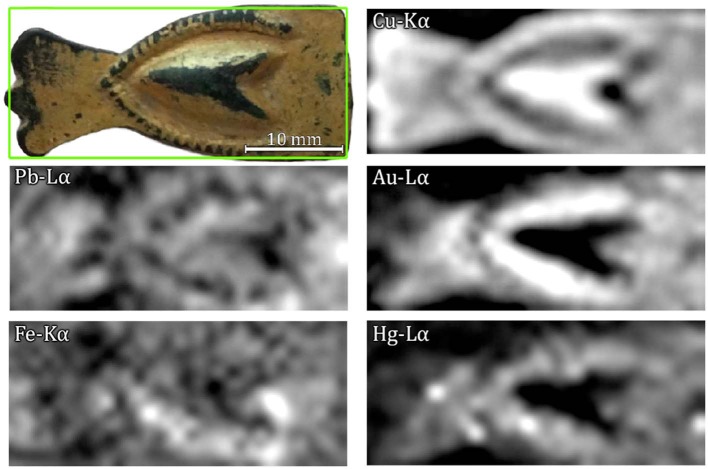
Analyzed sample (top left) and MA-XRF elemental distribution maps of lead L_α_, iron K_α_, copper K_α_, gold L_α_, and mercury L_α_.

Figure 2 reports the elemental maps distribution acquired by the MA-XRF scanning system. The elemental distribution maps show mercury correlated to gold. The simultaneous and correlated presence of both elements are an indicative that mercury and gold are mixed in the uppermost, gilded layer. Although MA-XRF information cannot be used to distinguish fire-gilding from cold-gilding, it certainly can strongly suggest the use of a mercury-gilding technique. This was also confirmed by previous SEM analysis performed by Ingo et al. (2018), which demonstrated that this gilded object was produced by fire-gilding technique.

The elemental maps also show a rather inhomogeneous distribution of certain elements, with a higher concentration of lead in the rightmost part of the sample and of iron in the lower part.

**Figure 3:**
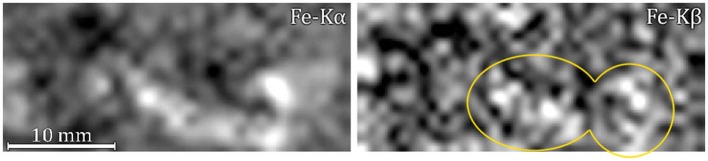
Elemental distribution maps of iron K_α_ **(left)** and iron K_β_ **(right)**.

The explanation for the latter may be three-fold: (a) the tube-sample-detector geometry—being the detector on the left-hand side of the tube—causing an overestimation of iron peaks or an increase in Cu-escape peaks (Trojek, 2011), (b) the preferential deposition of iron impurities during the burial period in the lower-concave region, or (c) a deposition of iron in both concave regions during the burial period combined with the geometrical factor. Due to the central relief in the sample, the combined effect described in (c) results in an increase in the signal from the lower concave region and a lower signal from the upper concave region. The elemental distribution map of iron is better represented in Figure 3, where a comparison between iron-K_α_ and iron-K_β_ maps is shown. It can be observed that the signal contribution is due mostly to the K_α_-line (maximum net area of 242 counts against 55 from K_β_), meaning that iron is not present as an alloying element but as an impurity deposited in the concave parts of the sample probably during the burial period. Furthermore, the characteristic low signal of alloy impurities detected by XRF would be suppressed by the gold layer deposited on the surface. Moreover, concentrations of secondary metals, such as iron and aluminum, appear frequently in corroded bronzes that have been buried for prolonged periods of time (Nørgaard, 2017).

For what concerns the lead presence, a lead segregation can occur if its content within the copper alloy exceeds 10 wt.% (Quaranta et al., 2014). On the other end, casting conditions, can play a significant role in the lead segregation/precipitation. In fact, lead and copper have significantly different melting temperatures and if the cooling rate is fast enough, the phase-diagram is disturbed and lead precipitates into a different phase (Callister, 2007). Moreover, both lead-L_α_ and -L_β_ elemental distribution maps are identical and with reasonable maximum net areas of 227 and 124, respectively, being indicative that the lead signal comes indeed from underneath the gilding layer.

**Figure 4:**
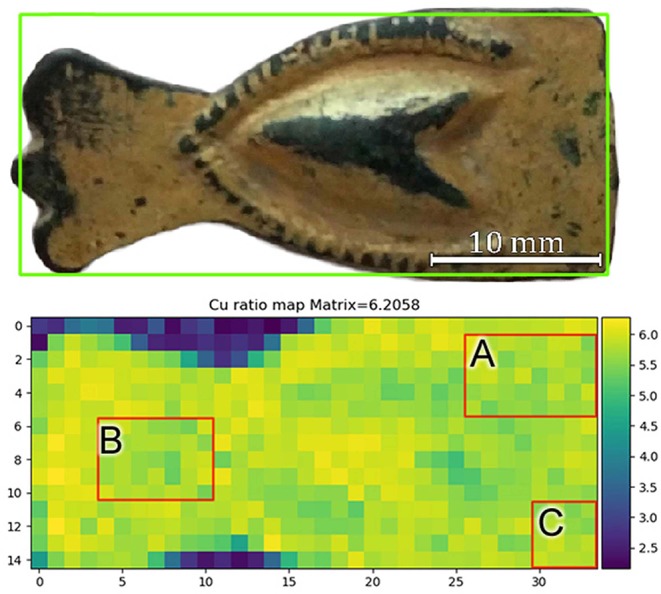
Selected areas for calculating the gilding layer thickness.

Since inhomogeneities and contaminations were detected in the sample, some areas of interest (AoIs) shown in Figure 4 were selected to well-define the areas where to apply the differential attenuation method. This was done in order to avoid the presence of lead islands and areas with higher iron signal for the calculation of the gilding layer thickness. The dataset is then restricted to 91 pixels out of the 310 pixels where gold was detected (Figure 4).

Amalgam-gilded artifacts can undergo corrosion (Ingo et al., 2016, 2018), as opposed to what is normally expected from golden objects. Micro-pores and fissures that remain in the finished gilded surface still allow the interaction between copper and corroding species from the environment (Ingo et al., 2018). The intermediate corrosion product layer created by this process and between the gold surface and the bulk metal can vary in thickness (Robbiola et al., 1998). In Equation (5) for the thickness calculation, the thick sample ratio [(I_Kα_/I_Kβ_)_thick_] is used acting as a calibration factor and it was determined by measuring six spots in the central portion of the artifact, where no gold was observed (Figure 2). This thick ratio supposedly represents the substrate directly in contact with the overlapping gilded layer, and the average measured value obtained was 6.206.

The amount of residual mercury present in the finished gilded layer can vary from 25 wt.% (near the ζ-phase α′-phase border in the Au-Hg phase-diagram) to 0.05 wt.% under a 600°C heating. However, a finished layer will usually retain about 8–25% of mercury (Anheuser, 1997). This amount varies according to the temperature used in the firing step, the initial mercury concentration (about 80–90 wt.%) and the thickness of the layer applied prior firing (Anheuser, 1997; Ingo et al., 2013). Gold and mercury have very close atomic numbers (92 and 93, respectively) but have a slight discrepancy in density (19.28 and 13.53 g/cm^3^, respectively). Variations in the mercury content present in the alloy (±10%) induce changes in the attenuation coefficients difference (2–3%) that are much less than the observed statistical uncertainty. Therefore, to estimate the attenuation coefficient for the K-lines of copper [μL variables in Equation (5)] of the gold layer, a chemical composition of 88 wt.% Au and 12 wt.% Hg was simulated and attributed to the layer (Table 1).

**Table 1:** Variations in the linear attenuation coefficients with varying quantities of mercury and gold.

**Hg (%)**	**μ_L_(Cu-K_a_) (cm^−1^)**	**μ_L_(Cu-K_β_) (cm^−1^)**	**–μ_L_(Cu-K_a_) + μ_L_(Cu-K_β_) (cm^−1^)**
2	3,930	3,038	−892
4	3,909	3,021	−887
6	3,888	3,005	−882
8	3,867	2,989	−877
10	3,846	2,972	−873
12	3,824	2,956	−868
14	3,803	2,940	−863
16	3,782	2,923	−858
18	3,761	2,907	−853
20	3,739	2,890	−848
22	3,718	2,874	−844

**Figure 5:**
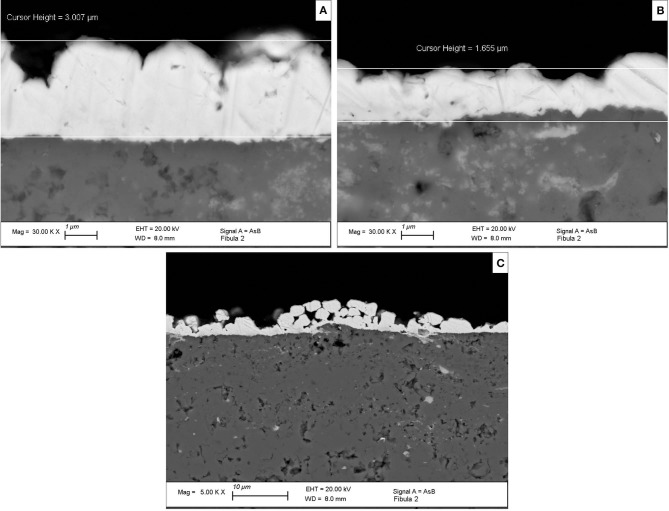
SEM-EDS images from sample's cross section reporting different gilding thicknesses ranging from 3 μm **(A)** to 1.6 μm **(B)**. A general overview of the gilding layer on top of a corrosion substrate presenting a high porosity and variable thickness **(C)**.

As previously stated, fire-gilded objects are usually characterized by the presence of several micro-pores and fissures. In Figure 5, SEM-EDS analysis of the sample cross-sections is reported. A highly fissured bright layer on top of a darker region (corrosion product layer) can be observed in Figure 5C. The presence of fissures drastically reduces the density of the superimposing gold layer (Ager et al., 2017; Giurlani et al., 2019) and if not taken into account may lead to misleading conclusions in regard of thickness calculation. As the method used in the study proposes a non-invasive approach, the fissure percentage estimated to account for the fissure influence was calculated through a simple image analysis of top-view (backscattered) SEM images, comparing the difference between the bright (gold) and dark (copper) pixels.

**Figure 6:**
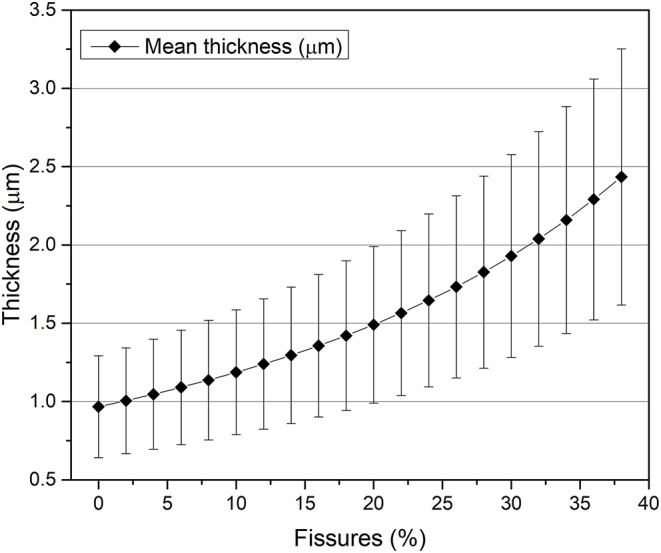
Mean thickness variation with increasing fissure volume.

The estimated fissure value was fed into the algorithm to correct the attenuation coefficients and density of the gold layer, simulating the gold *stratum* as a homogeneous mixture of air and gold-mercury alloy. Figure 6 shows an exponential increase in both standard deviations and mean values obtained when fissure percentage increases. The joint effect of substrate composition thickness variation (corrosion) and varying fissure volumes greatly contribute to a high standard deviation. In this way, as the mean thickness values may fall within a similar region, the direct influence of fissures over the mean calculated thickness cannot be directly measured. Nevertheless, a large standard deviation is not unreasonable. The gilding layer presents an inhomogeneous thickness distribution as demonstrated by the SEM-EDS images (1.6 and 3 μm) (Figures 5A,B).

The minimum detectable thickness can be calculated by the following relation:

(6) $$\begin{array}{l} \Delta d\;=\;\frac{\sqrt{1+\left(\frac{I_{K_{\alpha }}}{I_{K_{\beta }}}\right)}}{\sqrt{I_{K_{\alpha }}}}\frac{sin\;\Psi_{2}\;}{-\mu_{L}\left(E_{K_{\alpha }}\right)+\mu_{L}\left(E_{K_{\beta }}\right)} \end{array}$$

**Figure 7:**
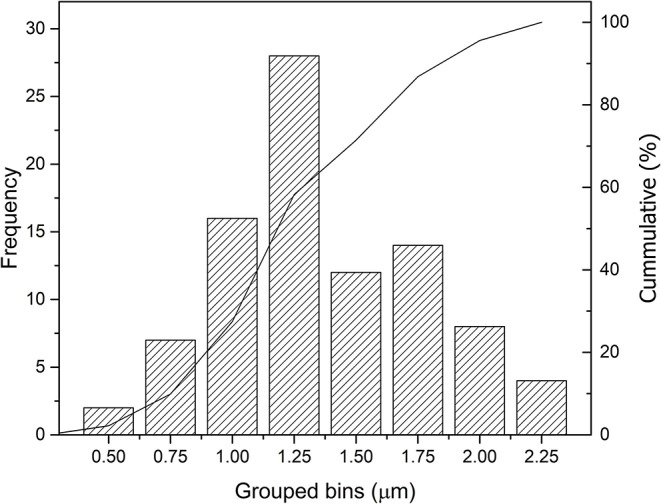
Grouped bins histogram of mean thickness values obtained with all 91 sampled pixels from the limited region of interest.

The copper Kα/Kβ ratio statistical error is 2.5%, having a corresponding minimum detectable thickness of 0.26 (Equation 6), which is almost constant. The observed standard deviation in the AoI (0.43 μm) can be interpreted, being statistical, as the square root of the quadratic sum of the statistical contribution and the remaining sample variation. The latter being 0.32 μm for the AoI, whereas the statistical uncertainty is 0.25, slightly less. The measured error (surface roughness, solid angle, etc.) is part of the sample contribution ($\sigma_{\text{sample}}^{2}$). This is true because the measuring error comes mostly from the sample through geometrical factors. When calculating the same parameters for the entire region where gold is found (310 pixels), a larger observed standard deviation is found (0.42 μm), demonstrating that the selected AoI is in fact more homogeneous in respect to the whole sample.The histogram of the calculated thickness distribution for the AoI (91 pixels) is shown in Figure 7 and, for the selected fissure percentage (12%), the gilded layer thickness mean value obtained was of 1.24 ± 0.43 μm. There is no significant variation between the individual thickness means of the three sampled areas (1.26 ± 0.43, 1.20 ± 0.42, and 1.25 ± 0.39). In addition, it is observed that the histogram shows a normal distribution that supports the statement that the measured mean value looks unbiased.

**Figure 8:**
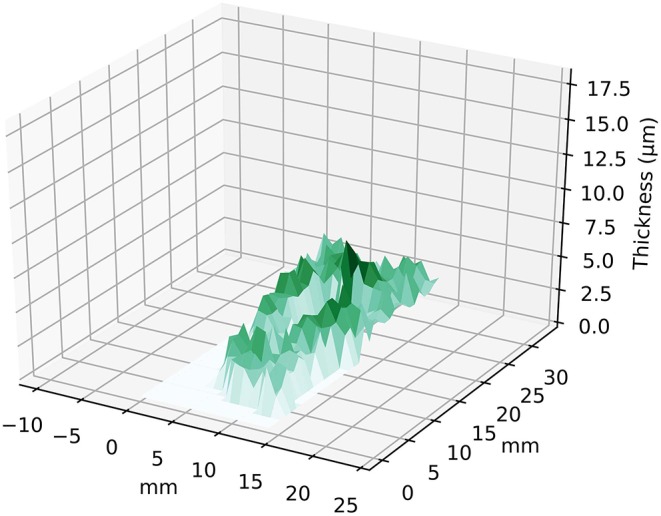
3D plot of mean thickness values obtained for the entire sample region.

In Figure 8 the 3D plot of thickness distributions for the entirety of the gold layer is shown. When performing the calculations over the entire range where gold is detected (310 pixels), the results present systematic errors, where mean thickness values of up to 6.24 μm were observed. The high values are obtained exclusively in the regions where iron or lead are present in higher quantities, demonstrating the strong influence caused by great sample inhomogeneity.

## Conclusions

In the overall framework of recent research developments in MA-XRF analysis, which started in the last decade (Dik et al., 2008), an attempt was made to evaluate the capacity of measuring thin metallic layers deposited on a sample surface. For the first time, a new algorithm applied to MA-XRF datasets was developed to automatically extract net peak areas and, at the same time, to non-invasively determine the average thickness of an area of a given superimposing layer. The innovative algorithm was able to account different layer chemical compositions and fissure volumes, recalculating the linear attenuation coefficient according to the user input.

The results obtained with the proposed methodology were confronted with SEM-EDS cross-section analysis to assess its reliability. The non-invasive approach suggested a mean thickness value of 1.24 ± 0.43 μm, with a maximum of 2.20 μm for the amalgam-gilded layer, whereas the latter pointed to values of 1.65 and 3.00 μm in two different analyzed regions.

Some parameters needed to be considered and cautiously studied to achieve the most accurate results possible. Small variations in the gold and mercury concentration within the binary (Au/Hg) gilding layer were found to not significantly affect the mean thickness results. This is shown by the fact that the changes in the value measured in different areas of interest do not vary between them. Nonetheless, considering the influence of fissures percentage in the superficial gilding layer demonstrated to be of fundamental importance as it influences exponentially the final results. Last, the substrate in direct contact with the studied gilding layer had to be considered homogeneous throughout the sample. Because of that, areas with observable inhomogeneities or considerably different from the exposed central relief had to be unconsidered by the algorithm to not bias the final results.

The larger dataset naturally provided by MA-XRF scans yielded greater statistics than traditional spot measurements and in a faster fashion, thus making it possible to obtain a more accurate mean value as shown by the histogram distribution. Moreover, MA-XRF scans and the elemental distribution maps provided crucial information for selecting suitable regions where to perform the thickness calculations.

Finally, the indirect determination of a mean thickness value for an ancient amalgam-gilding *stratum* in a total non-invasive manner that is satisfactorily close to results obtained through other destructive methods was demonstrated to be possible.

## Data Availability Statement

The datasets generated for this study are available on request to the corresponding author.

## Author Contributions

SB drafted the work and performed the MA-XRF scanning experiments together with SR. SB, GG, and RC performed the scripting and MA-XRF data evaluation. GC, MA, and CR provided the sample for analysis, performed the sample treatment, sectioning, and SEM-EDS analysis. AF and PB developed a custom electronic readout for the MA-XRF scanner. LT conceptualized and reviewed the paper.

### Conflict of Interest

SR was employed by the company Ars Mensurae srl. The remaining authors declare that the research was conducted in the absence of any commercial or financial relationships that could be construed as a potential conflict of interest.
